# An Additive Manufacturing Method Using Large-Scale Wood Inspired by Laminated Object Manufacturing and Plywood Technology

**DOI:** 10.3390/polym13010144

**Published:** 2020-12-31

**Authors:** Yubo Tao, Qing Yin, Peng Li

**Affiliations:** 1 State Key Laboratory of Biobased Material and Green Papermaking, Qilu University of Technology, Shadong Academy of Sciences, Jinan 250353, China; taoyubo@qlu.edu.cn (Y.T.); qluyinqing@163.com (Q.Y.); 2 College of Material Science and Engineering, Northeast Forestry University, Harbin 150040, China

**Keywords:** veneer, laser-cut, additive manufacturing, wood composite

## Abstract

Wood-based materials in current additive manufacturing (AM) feedstocks are primarily restricted to the micron scale. Utilizing large-scale wood in existing AM techniques remains a challenge. This paper proposes an AM method—laser-cut veneer lamination (LcVL)—for wood-based product fabrication. Inspired by laminated object manufacturing (LOM) and plywood technology, LcVL bonds wood veneers in a layer-upon-layer manner. As demonstrated by printed samples, LcVL was able to retain the advantageous qualities of AM, specifically, the ability to manufacture products with complex geometries which would otherwise be impossible using subtractive manufacturing techniques. Furthermore, LcVL-product structures designed through adjusting internal voids and wood-texture directionality could serve as material templates or matrices for functional wood-based materials. Numerical analyses established relations between the processing resolution of LcVL and proportional veneer thickness (layer height). LcVL could serve as a basis for the further development of large-scale wood usage in AM.

## 1. Introduction

**Figure 1:**
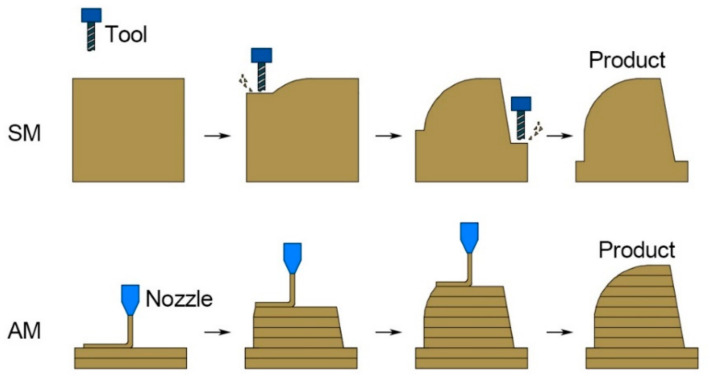
Subtractive manufacturing (SM) and additive manufacturing (AM).

**Figure 2:**
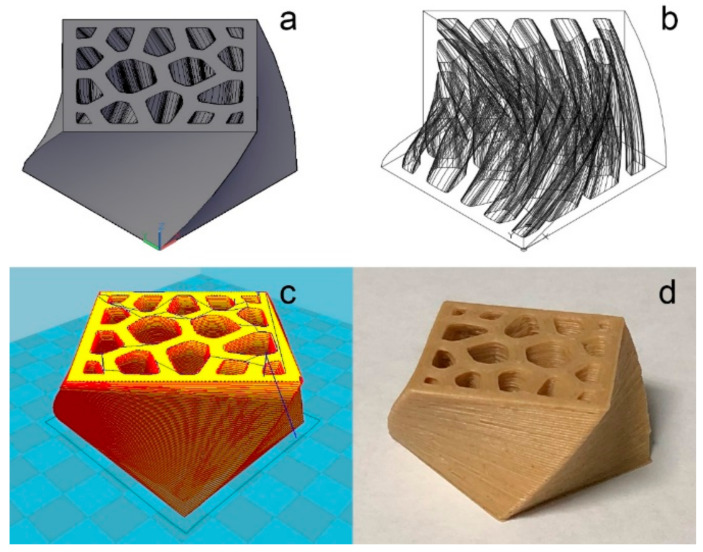
An illustration of the typical AM process. (**a**) 3D model of the desired product; (**b**) “wireframe” display of the desired product; (**c**) model after slicing into layers by the CURA software; (**d**) desired product constructed using fused deposition modeling (FDM) 3D printing.

The controlled process of material removal is a definitive trait of subtractive manufacturing technologies (SM). Traditional wood-processing techniques such as sawing, milling, turning, carving, and grinding, as well as relatively modern techniques such as CNC (Computer Numerical Control), are all categorized as SM [1]. As shown in Figure 1, portions of the raw material are methodically removed until the intended shape is achieved. By contrast, additive manufacturing (AM), as shown in Figure 1, often referred to as 3D printing, is a process of joining materials, typically in a layer-upon-layer manner, in accordance with three-dimensional (3D) model data [2]. Fabrication using AM begins with a 3D model of the desired product, such as the model shown in Figure 2a,b. Subsequently, 3D printing software will slice the model into horizontal cross-sectional layers, as shown in Figure 2c. Ultimately, the model is fabricated by stacking layers, an example of which is shown in Figure 2d.

Variations of AM are differentiated by their respective layer-fabrication techniques, including stereolithography apparatus (SLA), fused deposition modeling (FDM), laminated object manufacturing (LOM), selective laser sintering (SLS), and direct energy deposition (DED) [3]. Notably, AM is especially advantageous compared to SM when manufacturing products with exceptional geometric complexity. Currently, AM technologies have extended to areas in the aerospace, automotive, medical, architecture, and fashion industries [4]. The continuously increasing demand for renewable and sustainable products sourced from petroleum-free and carbon-neutral origins has driven the development of novel materials for AM methods in recent years.

Wood derivatives, such as wood flour and sawdust, as well as the components of wood, i.e., cellulose and lignin, are naturally abundant, biodegradable, biocompatible, and chemically modifiable materials that have shown promising potential for AM [5,6]. Existing research has shown that the practicability of incorporating wood-based materials in AM is largely dependent on the respective AM technique [7,8,9,10,11,12,13]. At present, layer fabrication techniques using wood-based materials may be divided into two general categories: extrusion-deposition and granular bonding. Extrusion-deposition fabrication primarily employs wood-plastic composite filaments that could be used in FDM [7,8]. In addition, studies have also shown that it is possible to extrude and deposit a slurry mixture of sawdust and adhesive directly to achieve similar AM results [9,10,11]. Likewise, granular bonding comprises two distinct variants. One type involves melting powdered mixtures of thermoplastic polymers and wood-based materials with high-intensity lasers [12], a technique utilized by SLS, whereas the other relies on the solidification reaction of a wood-based bulk material, as inorganic binders blend upon contact with water [13].

LOM is one of the first commercially available AM techniques, in which sheets of material, including metal, plastic, and paper, etc., are cut, often with lasers or mechanical cutters, to precisely resemble the shape of the cross-sections of the desired product. Successive layers are bonded layer upon layer until the object is completed [14,15]. Nevertheless, wood-based product fabrication with the aforementioned AM techniques is primarily dominated by micron scale powder and fiber materials. Current preparation methodologies not only increase the overall processing difficulty of wood-based materials, but also create drastic discrepancies, in both appearance and mechanical properties, compared with the original wood. 

The utilization of large-scale wood materials in AM has rarely been explored. Existing studies have investigated the application of one-dimensional wood-based materials, such as sticks and strips, in AM. For example, one study involved dispensing chopsticks coated in wood adhesive from a projection mapping-guided handheld stick dispenser to construct architectural structures [16]. Another study fabricated high-resolution timber structures with continuous willow withe-based solid wood filaments, a robotic fiber placement process, and topology optimization [17]. 

This paper proposes ideas for an alternative AM method for wood-based product fabrication that would be able to utilize large scale wood-based materials, such as wood veneer (a two-dimensional surface), by combining plywood technology with the basis behind LOM [18,19]. In addition to granular and strip-like, wood-based AM materials, the proposed method could enable the use of plate-like wood materials in AM. Furthermore, this study is characterized by the use of simple processing techniques, such as cutting and gluing, and AM characteristics to manufacture wood products with complex shapes and internal structures without advanced subtractive techniques, such as robotic CNC engraving. Moreover, its AM capabilities could be used for creating designable templates and material matrices for functional wood-based materials, such as sound absorbers and composites. Inspired by LOM, this process can be named laser-cut veneer lamination (LcVL), in which sheets of laser-cut veneer form cross-sectional layers that are bonded layer upon layer to form wood products with complex geometries and internal voids.

## 2. Materials and Methods

An LcVL-printed product was fabricated based on the design shown in Figure 2 to demonstrate the capabilities of the proposed AM method.

### 2.1. Modeling

**Figure 3:**
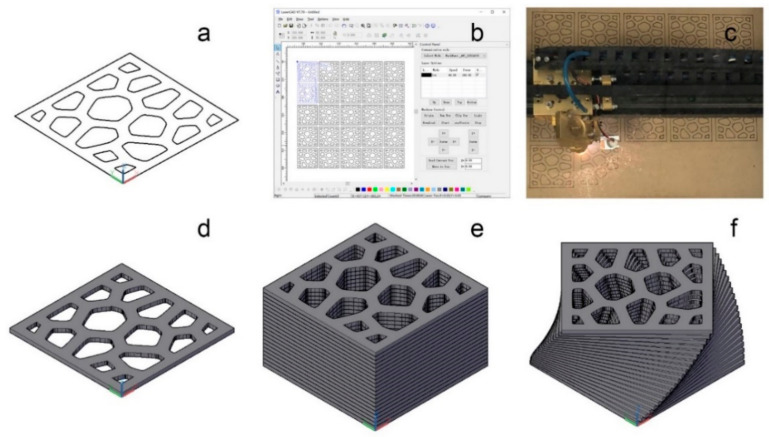
Model design methodology. (**a**) Outline of a Voronoi cellular-patterned cross-sectional layer; (**b**) Setting laser-processing parameters in the LaserCAD software, such as laser power, moving speed, etc.; (**c**) Laser-cut wood; (**d**) A layer slice after 1.5 mm of extrusion; (**e**) Stacking of layers along the Z-axis to create a layered model; (**f**) Layers are rotated to produce the model of the desired product.

The procedures used in the construction of a 3D model of the sample were as follows: as depicted in Figure 3a, a 50 mm × 50 mm square was created on the XOY plane (AutoCAD, student version 2019, San Rafael, CA, USA). The interior of this square was then partitioned into 16 Voronoi cells. An extrusion of 1.5 mm was applied to the surface along the Z-axis to create a layer model for the sample, as shown in Figure 3d. A total of 20 duplicates of the layer model were stacked along the Z-axis, as illustrated in Figure 3e. Lastly, all layers underwent rotation with the angle of rotation increment by 2.25° with each passing layer, as shown in Figure 3f. 

### 2.2. Processing

Poplar (*Aspen*) veneer with a nominal thickness of 1.5 mm and 8% moisture content was adopted in this work. The design shown in Figure 3a was fed to the LaserCAD software (Shenzhen Qiancheng Co., Ltd., Shenzhen, China) for setting laser processing parameters such as path, power, and speed. As shown in Figure 3c, a laser-carving machine (Model 4060, Huitian Laser Instrument Co., Ltd., Jinan, China) was used to cut veneers following the path and parameters set in Figure 3b to create each layer of the desired product. 

The top of each layer was coated with polyvinyl acetate (PVA) adhesive (Pattex 710, Pattex Co., Ltd., Shanghai, China) before being stacked to form a mat in accordance with the model design. A mold of the model contour could be used to guarantee layer placement precision. After 2 min of deposition, the mat was pressed for 5 min under 10 N using a small cold presser (lab-made) to complete the bonding process. ifferent adhesives could be used with adjusted pressing parameters.

## 3. Results and Discussion

### 3.1. The LcVL Product

**Figure 4:**
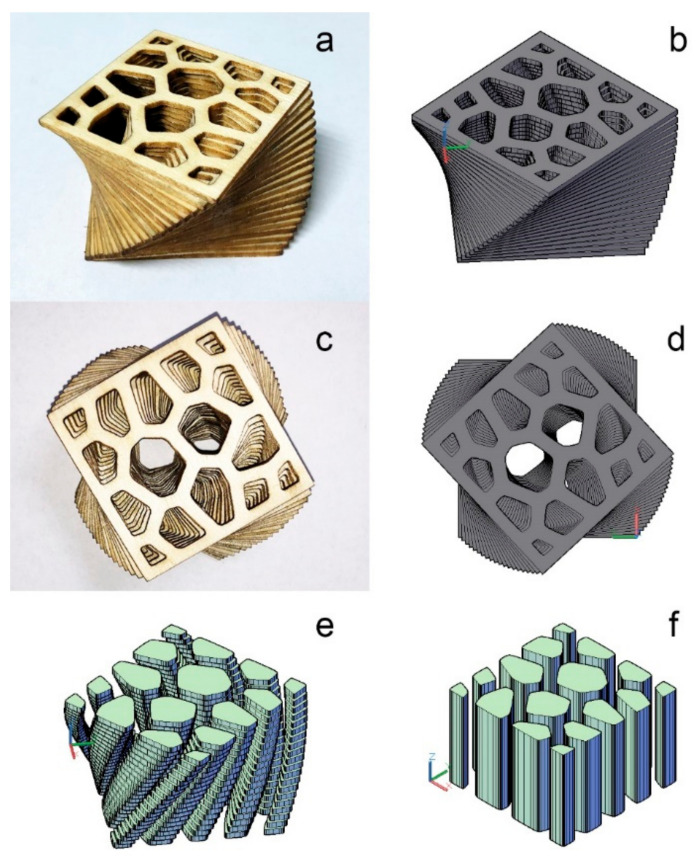
Comparison between the laser-cut veneer lamination (LcVL) product and its 3D model. (**a**) Orthographic view of the LcVL product; (**b**) Orthographic view of the 3D model of the product; (**c**) Top view of the LcVL product; (**d**) Top view of the 3D model of the product; (**e**) Tubular voids present in the 3D model of the product postrotation; (**f**) Tubular voids present in the 3D model of the product prerotation.

As shown in Figure 4, the LcVL product was fabricated by stacking and bonding wood veneers in a layer-upon-layer manner. The product demonstrated that the LcVL procedure was able to take advantage of the qualities of additive manufacturing, specifically, the ability to manufacture complex geometries, such as internal voids, that are nigh impossible to accomplish using SM techniques, such as CNC. However, since LcVL is based on LOM characteristics, although the overall product formation is additive in nature, the production of each layer via cutting is a subtractive process. These subtractive drawbacks should be marginal in comparison to the technical simplicity of the LcVL process. Residual materials could be repurposed as raw materials for 3D printing. For example, leftover veneers could be used to produce wood powder for wood/polylactic acid filaments. 

Furthermore, the surface area of tubular voids present in Figure 4e is 1.27 times the surface area of SM possible tubular voids in Figure 4f. The increased surface area in products with intricate geometrical structures, such as the product presented in Figure 4, could prove beneficial for the development of special-purpose, wood-based products. For example, the spacious tubular voids of complex LcVL-printed structures contain larger void surfaces and enable greater convenience for architecting desired tortuosity, which could improve sound absorption compared to standard SM possible structures [20]. Overall, as demonstrated by the printed product in Figure 4, LcVL was able to properly realize the 3D model of the desired product to a satisfactory degree. However, the LcVL method is not ideal for fabricating products with high angle overhangs without additional external support to ensure uniform pressure on each layer.

Notably, comparing the printed models present in Figure 4a (LcVL) and Figure 2a,d (FDM) revealed visible distinctions in processing resolution. As will be discussed in detail in the following section, the fabricating resolution of LcVL-printed products is primarily dependent on the layer parameters.

### 3.2. Effects of Layer Parameters on Processing Resolution

**Figure 5:**
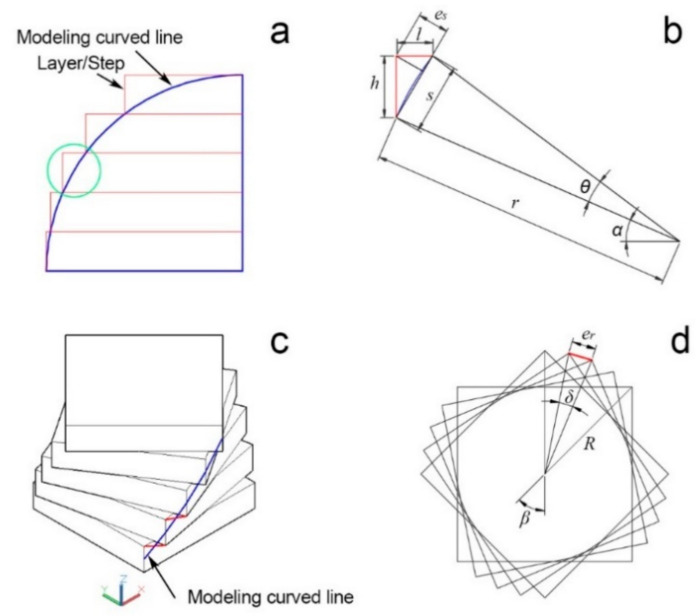
(**a**) Theoretical manufacturing error between contours of the modeling curve line and LcVL layer stacking; (**b**) Calculation parameters for the theoretical manufacturing error from LcVL layer stacking; (**c**) Theoretical manufacturing error between contours of the modeling curve line and postrotation LcVL layer stacking; (**d**) Calculation parameters for the theoretical manufacturing error from postrotation layer stacking.

As depicted in Figure 5a, LcVL is unable to replicate the modeling curve line (MCL) with perfect precision. The resulting step-like contour along the Z-axis comprises a theoretical manufacturing error (TME) between the 3D model and the fabricated product. Using the region circled in green in Figure 5a as an example, the relation between layer (veneer) height and TME could be described as follows: 

As shown in Figure 5b, when s is the arc length of a MCL, r the radius of the MCL, θ the central angle of the MCL, α the angle of the MCL to horizontal, h the layer height, and $e_{s}$ the TME from layer height, then

(1) $$s=2r\sin\frac{\theta }{2}$$

(2) $$l=\sqrt{s^{2}-h^{2}}$$

(3) $$\frac{1}{2}s\cdot e=\frac{1}{2}h\cdot l$$

(4) $$e_{s}=\frac{h\cdot \sqrt{s^{2}-h^{2}}}{s}$$

**Figure 6:**
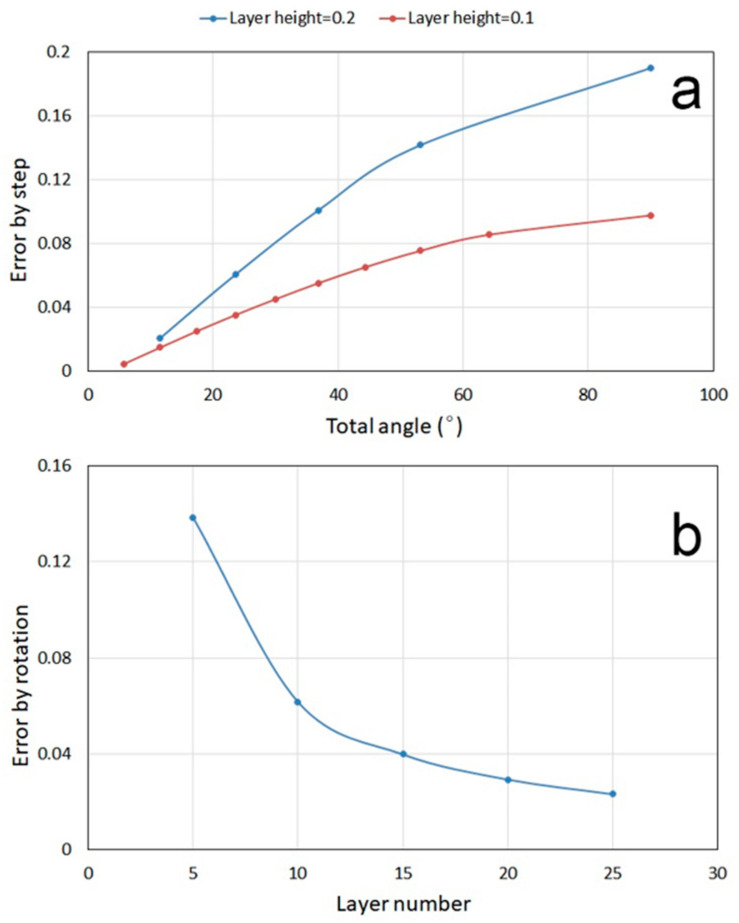
(**a**) Relation between layer height and theoretical manufacturing error (TME) from layer height, where total angle is the sum of *α* and *θ*; (**b**) Relation between layer number and TME from layer rotation.

The relation between layer height and TME from each step/layer of a quarter circle MCL with radius 1 was calculated and plotted in Figure 6a. The quarter circle was divided into five and ten layers to obtain proportional layer heights of 0.2 and 0.1, respectively. As can be seen in Figure 6a, the proportional layer height of 0.2 consistently exhibited greater TME compared to the smaller layer height of 0.1. Therefore, TME is positively associated with layer height.

In addition to the TME caused by layer height, the fabrication accuracy of the LcVL product in Figure 4 suffered further TME from layer rotation. As shown in Figure 5c, the apparent discrepancy between the MCL (highlighted in blue) and the printed product contributed to additional TME (highlighted in red). The TME from layer rotation could be described as follows:

As shown in Figure 5d, when R is the radius of rotation, n is the number of layers (layer number), β is the angle of rotation between the top and bottom layers, δ is the angle of rotation between successive layers, and $e_{r}$ is the TME from layer rotation, then

(5) $$\delta =\frac{\beta }{n-1}$$

(6) $$e_{r}=2R\sin\frac{\delta }{2}$$

(7) $$e_{r}=2R\sin\frac{\beta }{2\left(n-1\right)}$$

The relation between layer numbers (5–25) and TME from layer rotation for a height-1 hypothetical model with 45° of rotation between the top and bottom layers was calculated and plotted in Figure 6b. As can be seen in Figure 6b, the corresponding TME from layer rotation underwent reduction with larger layer numbers. Therefore, for the same product height, larger layer (veneer) numbers could result in decreased TME not only from layer rotation, but also from layer height as a result of the smaller layer height. Notably, for the product presented in this study (Figure 4), calculations showed that a 100% increase in layer number could increase the bonding area by 225%, which could increase production costs. The lower the layer height, the smaller the veneer thickness, which also increases the difficulty of veneer manufacturing. Notably, although the sample created for this study was a small object in the centimeter scale, the core characteristics of the LcVL method could be scaled up to manufacture structures in the meter scale, in theory. Naturally, corresponding parameters, such as the product height, layer height, and layer number should be adjusted accordingly to optimize the TME. 

### 3.3. Wood Texture Direction and LcVL-Product Structure

**Figure 7:**
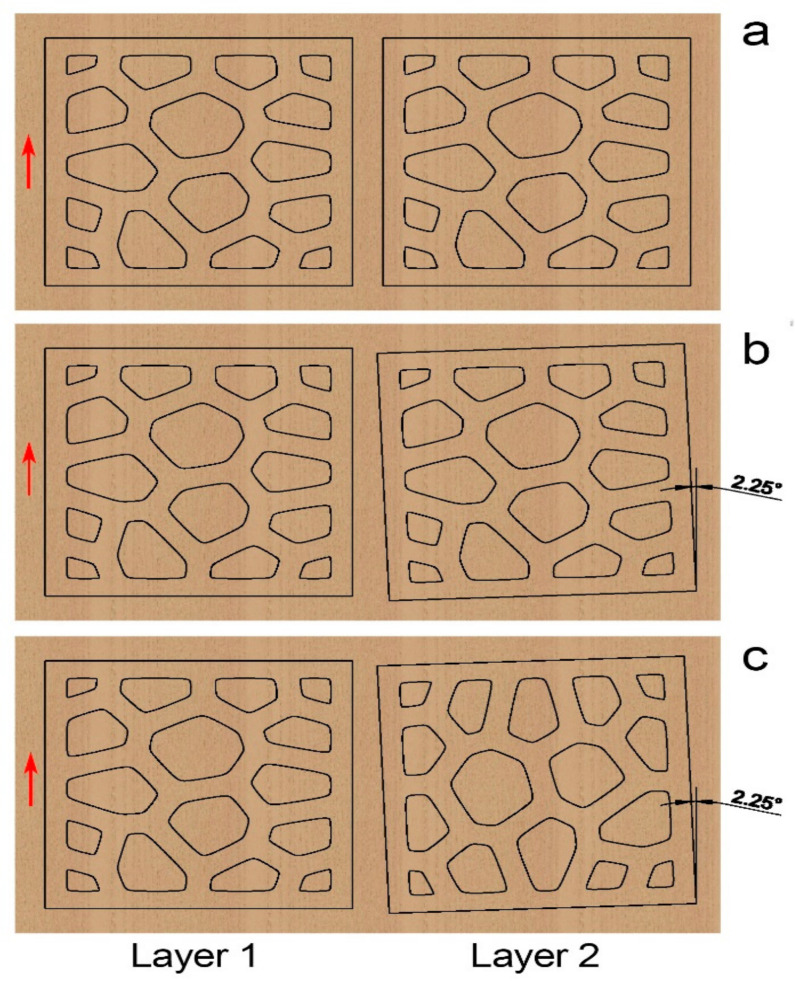
Wood-texture direction (indicated by red arrows) and distinct layer-cutting solutions (**a**) Cutting solution with identically cut layer 1 and layer 2. The resulting wood texture directions of the structure are 2.25° between each layer. (**b**) Cutting solution that produces a product with a consistent wood texture direction. (**c**) Cutting solution that produces a product with orthogonal wood texture directions between layers.

The structural directionality of LcVL products could be designed through wood texture directions. The sample presented in Figure 4 was created by stacking identical layers with each layer rotated by 2.25°. As shown in Figure 7a, a pair of identically-cut layers share the same wood texture direction. Thus, since all layers are 2.25° offset from their adjacent layers, the wood texture directions of all layers are 2.25° apart in this sample. However, as shown in Figure 7b, if layer 2 was cut with a counterclockwise 2.25° rotation from layer 1, then the wood texture direction of layer 2 would be 2.25° clockwise from layer 1. Thus, if such layer pairs were laminated together with a 2.25° counterclockwise layer-to-layer increment, the resulting product would have consistent wood texture direction. Alternatively, if layer 2 was cut with a 90° counterclockwise plus 2.25° counterclockwise rotation, as shown in Figure 7c, then the wood texture directions of layers 1 and 2 would be orthogonal in a product with 2.25° counterclockwise-rotated layers. The directionality of such a product could be analyzed with the orthogonal principle of plywood technology. The designability of LcVL-product structures is essential for creating material templates and matrices for composites of varying properties with LcVL.

## 4. Conclusions

LcVL is a relatively simple procedure for constructing customized and geometrically complex wood products which would otherwise be impossible, difficult, and/or costly using SM. Aside from raw material costs, costs of material waste and time consumption are optimizable factors of efficiency. In addition, the LcVL produce should be fairly adoptable, as its core technologies, laser-cutting and plywood, are already widely used in the wood industry.

From the above findings, the following conclusions could be made:

(1) As a combination of plywood technology and LOM, the LcVL method is a viable AM method which is capable of producing wood-based products with complex geometries and internal voids using large scale wood-based materials, specifically wood veneer.
(2) LcVL products have designable structures (complex internal voids and wood texture directions). Their designability could be used for creating material matrices and templates for functional wood-based materials, such as sound absorbers and composites.
(3) The LcVL method encountered more theoretical manufacturing errors compared to other AM techniques due to its use of larger scale raw materials with larger layer heights. Nonetheless, the LcVL method may be used for large scale wood materials with sufficient layer thickness and number.
(4) LcVL products could benefit greatly from postprocessing, such as surface finishing, for theoretical manufacturing-error reduction. In comparison with other AM techniques, larger amounts of wood and less adhesives are involved during the fabrication. The LcVL method could serve as a basis for the further development of veneer usage in AM.

## Data Availability

Data sharing not applicable.

## References

[1] Wimmer R., Steyrer B., Woess J., Koddenberg T., Mundigler N. (2015). 3D printing and wood. Pro Ligno.

[2] (2012). ASTM F2792-12a. Standard Terminology for Additive Manufacturing Technologies.

[3] Ngo T.D., Kashani A., Imbalzano G., Nguyen K.T.Q., Hui D. (2018). Additive manufacturing (3D printing): A review of materials, methods, applications and challenges. Compos. B Eng..

[4] Gardan J. (2016). Additive manufacturing technologies: State of the art and trends. Int. J. Prod. Res..

[5] Douglas G., Wang L., Wang J. Additive Manufacturing of Wood-Based Materials for Composite Applications. Proceedings of the SPE Automotive Composites Conference & Exhibition.

[6] Li T., Aspler J., Kingsland A., Cormier L.M., Zou X. (2016). 3D printing–a review of technologies, markets, and opportunities for the forest industry. J. Sci. Technol. Prod. Process..

[7] Tao Y., Wang H., Li Z., Li P., Shi S.Q. (2017). Development and application of wood flour-filled polylactic acid composite filament for 3d printing. Materials.

[8] Zhao X., Tekinalp H., Meng X., Ker D., Benson B., Pu Y., Ragauskas A.J., Wang Y., Li K., Webb E. (2019). Poplar as biofiber reinforcement in composites for large-scale 3D printing. ACS Applied. Bio. Mater..

[9] Gardan J., Nguyen D.C., Roucoules L., Montay G. (2016). Characterization of wood filament in additive deposition to study the mechanical behavior of reconstituted wood products. J. Eng. Fiber Fabr..

[10] Kariz M., Sernek M., Kuzman M.K. (2016). Use of wood powder and adhesive as a mixture for 3D printing. Eur. J. Wood Wood Prod..

[11] Rosenthal M., Henneberger C., Gutkes A., Bues C. (2018). Liquid Deposition Modeling: A promising approach for 3D printing of wood. Eur. J. Wood Wood Prod..

[12] Zeng W., Guo Y., Jiang K., Yu Z., Liu Y., Shen Y., Deng J., Wang P. (2013). Laser intensity effect on mechanical properties of wood–plastic composite parts fabricated by selective laser sintering. J. Thermoplast. Compos..

[13] Henke K., Treml S. (2013). Wood based bulk material in 3D printing processes for applications in construction. Eur. J. Wood Wood Prod..

[14] Feygin M., Pak S.S. (1999). Laminated Object Manufacturing Apparatus and Method. U.S. Patent.

[15] Wimpenny D.I., Bryden B., Pashby I. (2003). Rapid laminated tooling. J. Mater. Process. Technol..

[16] Yoshida H., Igarashi T., Obuchi Y., Takami Y., Sato J., Araki M., Miki M., Nagata K., Sakai K., Igarashi S. (2015). Architecture-scale human-assisted additive manufacturing. ACM Trans. Graph..

[17] Dawod M., Deetman A., Akbar Z., Heise J., Böhm S., Klussmann H., Eversmann P. (2020). Continuous Timber Fibre Placement. Impact: Design with All Senses.

[18] Eltawahni H.A., Rossini N.S., Dassisti M., Alrashed K., Aldaham T.A., Benyounis K.Y., Olabi A.G. (2013). Evaluation and optimization of laser cutting parameters for plywood materials. Opt. Laser Eng..

[19] Kubovský I., Kačík F. (2014). Colour and chemical changes of the lime wood surface due to CO_2_ laser thermal modification. Appl. Surf. Sci..

[20] Errico F., Ichchou M., De Rosa S., Franco F., Bareille O. (2020). Investigations about periodic design for broadband increased sound transmission loss of sandwich panels using 3D-printed models. Mech. Syst. Signal..

